# Breast clinic and life style study BLLISS

**DOI:** 10.1186/1477-7800-6-12

**Published:** 2009-06-30

**Authors:** Amtul R Carmichael, Laura Harbach, Richard Cooke

**Affiliations:** 1 Department of Surgery, Russell's Hall Hospital, Dudley, DY1 2HQ, UK; 2 Department of Audit, Russell's Hall Hospital, Dudley, DY1 2HQ, UK; 3 Department of Psychology, School of Life & Health Sciences, Aston University, Aston Triangle, Birmingham, B4 7ET, UK

## Abstract

**Background:**

Independent, strong and unequivocal evidence suggests that life style factors such as obesity and lack of physical activity along with certain reproductive choices can increase the risk of breast cancer. There are no studies measuring the effectiveness of guidelines from the Department of Health regarding life style choices made by women presenting to breast clinics. The aim of this audit was to study the prevalence of obesity, physical activity and reproductive factors in women referred to breast clinic.

**Patients and methods:**

All patients attending the Breast clinic as new referrals were invited to complete a life style questionnaire. The data was analysed for prevalence of various risk factors for breast cancer. Three hundred and 73 patients completed the questionnaire.

**Results:**

Final analyses of 373 patients demonstrated that 42% of women performed no exercise and only 24% of patients met Department of Health guideline of 30 minutes of exercise for 5 days a week. Overall 50% of patients were either obese or overweight and 22% of patients had BMI of > 30 kg/m^2^. The median age of menarche was 13 and 18% of women started their period below the age 12. Twenty one percent of women were nulliparous and 14% had their first live birth after the age of 30. Fourteen percent of patients were on the hormone replacement therapy of which 57% have used hormones for more than 5 years. Twenty two percent of women smoked and 9% of women consumed alcohol 5 days a week of which 13% had more than 4 glasses of alcohol in a day.

**Conclusion:**

There is preponderance of high risk life style choices in women attending breast clinic. If these life style options are not modified, there could potentially be a significant rise in the number of breast cancer in West Midlands.

## Introduction

Breast cancer is the most common cancer in women in the UK and is twice as frequent as any other female cancer. There has been a significant rise in the incidence of breast cancer in the last 20 years, as number of newly diagnosed breast cancer has increased from approximately 20,000 in 1988 to 45,000 in 2004. There is an understandable need to investigate and manage the cause of this rise in the incidence of breast cancer. The life style in women has significantly deteriorated in last 20 years [1]. Women are more likely to be obese, consume sub-optimum diet, have early menarche, have fewer children, breast feed less often and perform less physical activity. Most of these are modifiable risk factors for developing breast cancer.

The evidence that obesity contributes to the development of breast cancer in postmenopausal women is overwhelming and indisputable [2,3]. Obesity is estimated to cause 20% of all postmenopausal breast cancers and 27% of those in women > 70 years of age [4]. The increase in breast cancer risk attributed to obesity ranges 6–19% [5]. A 1-point gain in body mass index (BMI) is estimated to increase the risk of postmenopausal breast cancer by 3% [5], and every 5-kg increase in weight increases the relative risk (RR) of developing breast cancer in postmenopausal women by 1.08 [6]. It is shown that regular physical activity can prevent incidence and mortality of breast cancer [6]. Each additional hour of physical activity per week can decrease the risk of breast cancer by 6% (95% confidence interval = 3% to 8%) [7]. This risk decrease is even more marked in women who increased their recreational activity in their 50 s (27%) [8].

Recently, evidence has emerged which indicates a dose response between alcohol consumption and increased risk of developing breast cancer [9-13]. A meta-analysis of ninety-eight studies concluded that excess risk of developing breast cancer associated with alcohol drinking was 22% (95% CI: 9–37%); each additional 10 g ethanol/day was associated with risk higher by 10% (95% CI: 5–15%). Estimated population attributable risks was 6.0% in UK [11]. Alcohol is estimated to cause 4% of breast cancers in developed countries, and consuming one extra unit of alcohol per day increases the relative risk of developing breast cancer by 7% [10]. This level of risk remains even after controlling for potentially confounding factors (e.g. smoking, family history of breast cancer, race). Total alcohol intake of more than 27 drinks per week increases breast cancer risk in premenopausal women independently of the type of alcohol. Among postmenopausal women, an intake of spirits of more than six drinks per week increases breast cancer risk [14].

The aim of this study is to identify the life style choices being made by a cohort of women attending as a new patient to a Breast clinic in a large teaching Hospital.

## Patients and methods

All new patients attending breast clinic were invited to take part in this cross-sectional survey of life style and breast cancer at a large teaching hospital in the West Midlands, England between July 2008 to December 2008. The questionnaires were in SP15 format. Patients who were unable to read English were excluded. All male patients and follow up patients were excluded.

The patients were handed questionnaires at the visit before clinical consultation. The health care professional recorded the height, weight and BMI. The obesity was defined as WHO criteria. Women were questioned on their lifestyle choices such as smoking habits (cigarettes per day), alcohol intake (units per week) and level of physical activity (hours per week). For the purpose of this study, post-menopausal status was defined as 50 years of age or above. Women who had previously had a hysterectomy were categorised as post-menopausal.

The questionnaire was adopted from the clinical information that is collected as a part of new patient consultation. Because of the factual and basic nature of the information in the questionnaire, no formal validation was carried out.

Statistical methods Responses were analysed with frequencies, single variable and multiple response analyses using SPSS software programme™. Frequency table analysis and percentage distribution was carried out.

## Results

Over the 6 month period, 381 consecutive women Breast clinic as a new patients were invited to complete the questionnaire. One patient declined to complete the questionnaire and one did have her reading glasses. Three men were given the questionnaire and were not eligible for the study and four follow up patients completed the questionnaire. These patients were excluded from the study. Data presented here is for 373 patients.

### Reproductive factors

Median age of the patients was 43 years and 34% of patients were postmenopausal. The median age of Menache was 13 (range 9–18) in this cohort of patients and 18% of women has their first period below the age of 12. One-fifth (19%) of women were nulliparous and median age of birth of first child was 25 years (range 17- over 30 years of age). Almost one in six (14%) of women had first live birth after the age of 30. Over one third of women (38%) had a miscarriage or abortion. Fourteen percent of women took HRT, of whom 39% were on HRT for more than 5 and 18% took HRT for more then 10 years.

### Obesity and physical activity

**Figure 1:**
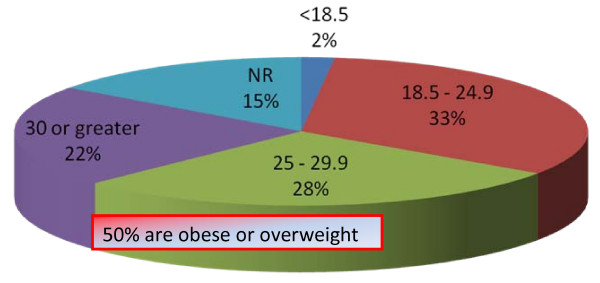
Almost half the patients presented to breast clinic were obese or overweight.

**Figure 2:**
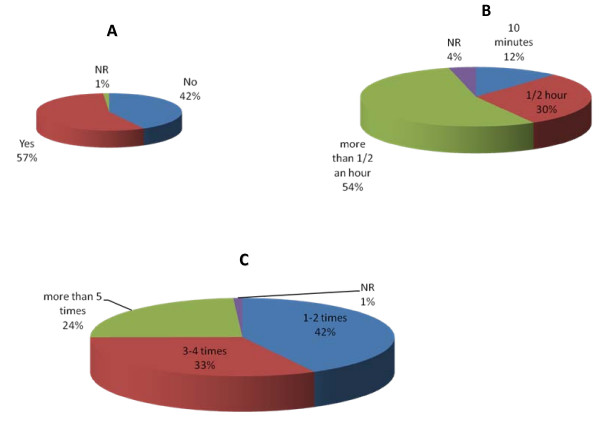
Only one third of patients attending breast clinic met department of health guidelines about physical activity.

In this cohort of patients, half the women had unhealthy weight. Almost 22% were obese and 28% were overweight Figure 1. Almost half the patients (42%) attending new breast clinic performed no regular recreational physical activity Figure 2. Of those who did regular exercise, only 14% exercised for 5 days or more per week and 31% of patients were regularly engaged in physical activity for more than 1/2 an hour Figure 2. Only 24% of women were meeting DoH guideline of 30 minutes of physical activity for 5 days a week.

### Alcohol intake and smoking

**Figure 3:**
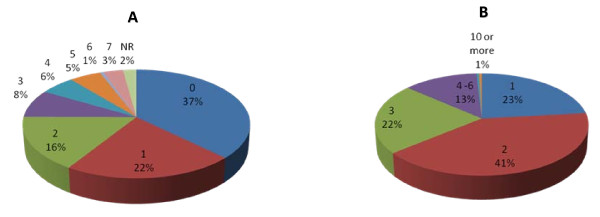
Most women presenting to breast clinic consumed alcohol in moderation and wine was most commonly used beverage.

The most frequent response among patients was that they did not consume alcohol at all (37%), while 22% of patients reported drinking on one day per week and 16% reported drinking on two days per week. Of the patients who reported consuming alcohol, 23% reported drinking one glasses, 41% reported drinking 2 glasses and 22% reported drinking three glasses. Wine was the most frequently reported type of alcohol consumed (64%) Figure 3a and 3b. Based on these questions it is not appropriate to estimate how many units of alcohol were consumed as we did not ask about the size of glass consumed, which will differ depending on type of alcohol, or the alcoholic volume of the drink consumed. Almost one fifth of women (22%) smoked.

Of the study cohort 5% were diagnosed with breast cancer. The numbers are too small for statistical analyses.

## Discussion

The risk factors for breast cancer can be divided into several main groups. Genetic or familial, reproductive or hormonal factors, lifestyle factors and environmental factors. The reproductive and life style factors are considered to be the modifiable risk factors for the development of breast cancer. The present study found that an alarming number of women attending for consultation as new patients were obese or overweight, about one third smoked and one in six were taking exogenous hormones. It was also found that a vast majority of women was not achieving the exercise guidelines set by the department of health. The Chief Medical Officer recommended a total of at least 30 minutes a day of at least moderate intensity physical activity on five or more days of the week for general health. [15]. The National Institute for Health and Clinical Excellence has recommended that primary care health providers should promote exercise with their patients [16].

The data that physical activity and maintaining a healthy weight improves the incidence of breast cancer is very powerful and supported by most long term, large cohort and Epidemiological studies. A systematic review of literature including 19 cohort studies and 29 case-control studies concluded that there was strong evidence for an inverse association between physical activity and postmenopausal breast cancer with risk reductions ranging from 20% to 80% [7]. A review of 34 case-control and 28 cohort studies concluded that this risk reduction was greater for postmenopausal healthy weight women [17]. Thus a substantial fraction of postmenopausal breast cancer may be avoided by changes in lifestyle later in life [18]. Despite long established and well reported association between lack physical activity and increased risk breast cancer, the pattern of life style choices of women in England and Wales should be a cause for concern. At present, a worrying 69% of women between 55–74 years of age have a sedentary lifestyle. In the present study 50% of postmenopausal women considered that they performed regular physical activity yet only 12% actually met DoH guidelines.

Among postmenopausal women in the UK, 5% of all cancers (about 6000 annually) are attributable to being overweight or obese [4]. Almost a quarter of the female population in England and Wales were found to be obese in 2006 according to the Annual Health Survey for England. The prevalence of obesity in the present study population was fairly representative of the UK population (28% overweight and 22% obese). The statistics released by the WHO in 2006 stated 30–40% of women in the post-menopausal age group in the UK were overweight and 25–31% were obese. It is estimated that 64% of women in postmenopausal group will be obese or overweight in 2010 (Forecasting Obesity to 2010). This will have worrying consequences for the incidence of breast cancer.

Epidemiological data suggest that increasing alcohol consumption by one unit of alcohol per day increases the relative risk of developing breast cancer by 7%. There was considerable diversity in the number of days women drank in a week, though there was a downward trend from the highest number reporting drinking on zero days per week, through to the lowest number reporting drinking on six or seven days per week. Most of this sample of women appear to be consuming alcohol at safe levels, with few women drinking daily and most women consuming little alcohol when they do drink. This pattern of alcohol consumption is unlikely to increase their risk of developing breast cancer, however it must be acknowledged that the responses were only collected on one occasion, and there may be variation in alcohol consumption that we have not accounted for. However, a vast majority (64%) of women who reported drinking, reported drinking wine, and this could be a cause for concern. Research by Gill and Colleagues [19,20] shows that people underestimate the number of units contained in a glass of wine. So, women could be putting themselves at greater risk by consuming more alcoholic units than they realise.

These data provide a snap shot of the life style behaviour of women presenting to breast clinic in Dudley and provide a clear idea of the extent of the modifiable risk factors in patients attending breast clinic. The weaknesses of this study are a relatively small sample size and potential for confounding variables. This is an observational study and relied on self-reporting. Nevertheless, these data suggest that women may be exposing themselves to increased risk of breast cancer by life style choices.

## Conclusion

In Dudley population, there is a preponderance of life style related risk factors that increase the risk of developing breast cancer. The health education message about physical activity and healthy weight is clearly not reaching this cohort of patients. These data point to trend that it is possible that patients in Dudley could be exposing themselves to higher breast cancer risk based upon our current understanding of the risk factors associated with the disease. More research is needed to investigate this life style behaviour in order to design effective interventions for life style modifications. These alarming statistics are likely to have significant impact on the incidence of breast cancer in women in West Midlands. If further rise in incidence of breast cancer is to be prevented then a more robust system of education is needed.

## Competing interests

The authors declare that they have no competing interests.

## Authors' contributions

ARC conceived the idea, designed the questionnaire and recruited the patients, LH collated and analysed the data and RC advised about alcohol intake data collection and wrote the section of the paper about alcohol intake.
